# Histopathological evaluation of bone formation using hydroxyapatite/β-tricalcium phosphate following tooth extraction: A comparative study of two time intervals

**DOI:** 10.34172/japid.025.3476

**Published:** 2025-11-10

**Authors:** Omid Moghaddas, Nima Naddafpour, Sareh Farhadi, Zahra Mosalmani, Sephora Khandan

**Affiliations:** ^1^Department of Periodontology, Dental Faculty, Islamic Azad Medical Sciences University, Tehran, Iran; ^2^Department of Oral and Maxillofacial Pathology, Dental Faculty, Islamic Azad Medical Sciences University, Tehran, Iran; ^3^Private Practice, Tehran, Iran; ^4^Private Practice, Los Angeles, California

**Keywords:** Alloplast, Alveolar ridge augmentation, Bone resorption, Bone transplantation, HA/βTCP

## Abstract

**Background.:**

Ridge resorption following tooth extraction can be managed with bone graft substitutes. The present study histologically evaluated the amount of new bone formation 3 and 4 months after tooth extraction and ridge preservation using the hydroxyapatite/β-tricalcium phosphate (HA/βTCP) graft material (OSTEON II).

**Methods.:**

Forty patients requiring tooth extraction and subsequent implant placement were enrolled in this randomized controlled clinical trial. Ridge preservation using HA/βTCP alloplast (OSTEON II) in extraction sockets was divided into three months (group A) and four months (group B). Bone samples were collected from each grafted socket at the implant placement site. Histomorphometric analyses were performed to determine the amount of new bone formation and the residual graft material. In addition, we evaluated changes in histological indices, i.e., inflammation rate, percentage of ossification, and the amount of remaining biomaterial.

**Results.:**

There were no significant differences in the amount of newly formed bone and the residual graft material between the two groups. In the 3-month group, an average of 20.11% of newly formed bone and 6.82% of the remaining graft were seen. In contrast, they were 20.67% and 7.38% in the 4-month group.

**Conclusion.:**

The findings suggest that the HA/βCP bone graft material (OSTEON II) may enhance bone regeneration within a shorter healing time.

## fIntroduction

 One concern following tooth extraction is the problems caused by the dimensional changes that occur afterward. This forces clinicians to perform reconstructive treatments and increase bone volume before implant procedures.^1^ Around 0.34–7.7 mm of resorption in ridge width and 0.2–3.25 mm of reduction in height occur 6–12 months after tooth extraction.^2^ This is the best time to preserve tooth socket dimensions.^3^

 Ridge preservation methods prevent 40–60% of alveolar bone atrophy following tooth extraction. Resorption typically occurs 2–3 months after tooth extraction and continues at a rate of 0.25–0.5% per year.^4^ Several experimental studies have evaluated using graft materials to increase the width or height of atrophic alveolar ridges or repair bone lesions. This method was first conducted by Boyne^5^ in 1970. It has gained relevance in recent years due to its high success rates. Because of the increasing demand for implant treatments, various materials and techniques have been developed to maintain the dimensions of extraction sockets, including allograft, alloplast, and xenograft particles.^6^ Placing implants is required in cases of severe resorption of the alveolar ridge dimension. Complex graft treatments are challenging.^7,8^ Autogenous bone from intraoral sources has been the gold standard for many years.^9^ However, it is less considered due to the need for secondary surgery at the donor site, invasiveness, and limited available bone volume. Synthetic materials have been used for many years due to their biological compatibility and good shelf life. Much research has been performed on animal specimens,^10-12^ unlike a few studies on human specimens, which have mostly focused on radiographic examination and the role and impact of graft materials that require histological examination. Ridge augmentation can help preserve extraction sockets and prevent progressive resorption.

 This study used hydroxyapatite/β-tricalcium phosphate (HA/βTCP) as a graft material (OSTEON II, manufactured by Dentium, Korea). Evaluations performed on synthetic grafts (biomaterial calcium phosphate) alone or in comparison with other grafts yielded different results regarding the amount of bone obtained. For this reason, given the use of synthetic grafts in animal specimens and their comparison with autograft bone, further studies are needed to evaluate the histological degradation rate of synthetic HA/β-TCP particles, the bone formation rate, and the percentage of biomaterial remaining in human specimens.

 Due to the long intervention period for tooth socket regeneration and the inconsistencies in various studies^10-13^ regarding the time required for proper bone formation (2, 3, and 4 months), the question is, “Is it possible to achieve the same success rate in bone formation in a shorter period (3 months) instead of 4 months?” Furthermore, histological studies have shown that the rates of bone formation at 2 and 4 months differ significantly.^13^ Therefore, due to the importance of time, this study evaluated the effect of HA/βTCP synthetic material at 3- and 4-month intervals on extraction socket bone formation.

## Methods

 This randomized controlled clinical trial (before and after) was approved by the Ethics Committee under the code IR.IAU.DENTAL.REC.1396,15 and IRCTID: IRCT20180714040460N2.

 Forty patients requiring the extraction of a single-rooted tooth and subsequent implant placement, with no indication for immediate implant placement, were selected from the Periodontology and Implant Department of the Faculty of Dentistry, Islamic Azad University, Tehran Branch. The sample size of 40 patients (n = 20 per group) was determined based on a previous clinical trial by Whetman and Mealey,^13^ which used at least 14 patients per group. It was calculated using power analysis to detect significant differences in new bone formation. To follow a similar structure and ensure clinical relevance, we selected 40 samples for comparison of 3- and 4-month healing intervals. Unlike Whetman and Mealey’s study,^13^ which used DFDBA, our study focused on HA/βTCP, and the design was exploratory in nature. Only intact extraction sockets with four bony walls were included in the study. Standard exclusion criteria for the bone grafting procedure were applied, including allergy, infectious diseases, systemic or local active diseases, and known medical or pharmacological conditions that alter soft tissue and bone repair (such as uncontrolled or poorly controlled diabetes mellitus and taking bisphosphonates and immunosuppressive drugs), pregnancy, and short-rooted or malpositioned teeth, in which core biopsy would result in involvement of the bony walls along the socket wall. After the study’s purpose was explained, the patients signed informed consent forms. Diagnostic procedures were performed to evaluate the extraction site: radiographic evaluation, impression-taking, preparing study casts, and clinical examination. After preparing the study casts, the stent was created as a fixed reference to determine the exact sampling location from the extraction socket. The HA/βTCP synthetic material with 500–1000-µm particles (manufactured by Dentium Korea under the OSTEON II brand) was used to graft the extraction socket.

###  Surgical procedure

**Figure 1:**
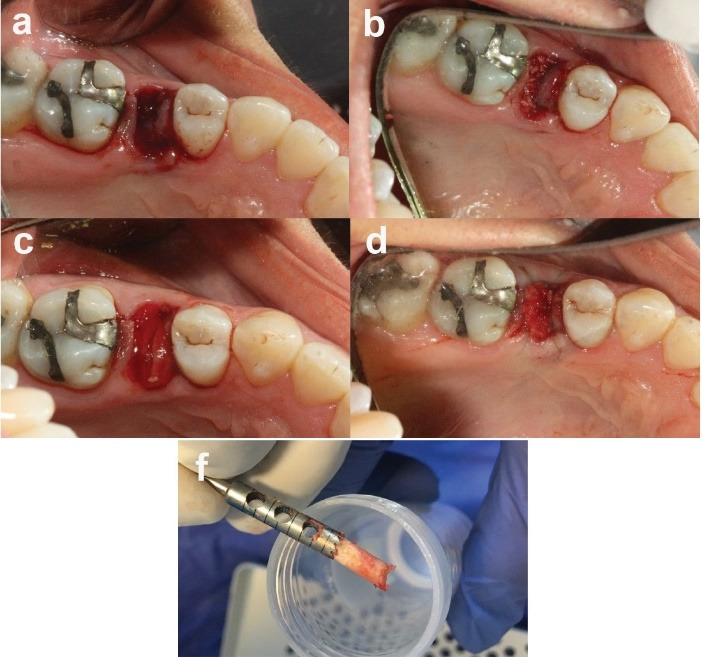
Clinical procedure: (a) atraumatic extraction of the tooth with hopeless prognosis, (b) graft placement in the socket, (c) collagen membrane application, (d) tension-free suturing (occlusal view), and (f) bone sampling using a trephine bur (2 mm inner and 3 mm outer diameter)

 Before surgery, the patients were randomly assigned to two groups using opaque envelopes (Figure 1). After debridement, atraumatic extraction was performed under local 1:80000 lidocaine, followed by irrigation and rinsing. The presence or absence of dehiscence and the number of bony walls were checked. A Williams probe was used to confirm the presence of mesial, buccal, distal, and lingual bone walls through sounding. The graft was hydrated using sterile saline for 10 minutes and then placed in the extracted tooth socket to ensure it was not overfilled. Extraction sockets were sealed with a collagen sponge (Ateloplug/Korea), and the area was sutured with a 5-0 nylon cross mattress suture.^14^

###  Postoperative procedure

 Antibiotic therapy was prescribed, consisting of 500 mg of amoxicillin (tid) for 7 days and mouth rinsing with 0.12% chlorhexidine twice daily for 30 seconds over 4 weeks. Patients allergic to penicillin were given 100 mg of doxycycline once a day for seven days. Postoperative pain was controlled with NSAIDs and opioid analgesics. Each patient was referred for a secondary surgical visit at the appointed time. A trephine bur with an inner diameter of 2 mm and an outer diameter of 3 mm was used to perform a core biopsy, sampling at a depth of at least 8 mm using a measuring stop. The bony samples were placed in a 10% neutral formalin buffer solution.^14^

###  Blinding the examiner 

 Each patient was assigned a specific code at the first appointment, and the biopsies were sent to the laboratory using that code. The examiner was unaware of the treatment groups and evaluated the results based on the codes.^14^ The study was double-blinded: both the evaluator and the individual performing the histological analysis were not aware of the group assignments.

###  Analysis and histological processes

 Core biopsies were collected using a trephine bur and placed directly in a 10% neutral formalin buffer. The cores were decalcified, dehydrated, and embedded in paraffin. Then, 4-µm-thick sections were prepared for histomorphometric examinations. Finally, the tissue was stained using conventional hematoxylin staining methods.^13^

 An oral pathologist examined the stained sections at × 100 magnification under a Nikon YS-100 light microscope with a graduated lens to determine the percentage of viable bone, the amount of residual biomaterial, and inflammation.^15^

## Results

 Forty patients, all males, were divided into groups A and B, with a mean age of 51.8 and 52.6, respectively. Twenty patients were recalled after 3 months, and the other 20 after 4 months. Following the surgical procedure, the samples were prepared and sent to the pathology laboratory to evaluate the effect of the particles. Table 1 shows the results obtained from 40 samples.

**Figure 2:**
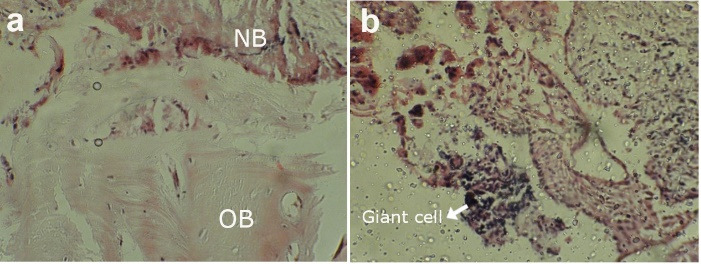
The histological analysis of the samples: (a) new bone formation (NB) versus old bone (OB), and (b) inflammation levels observed in the tissue

 Histological evaluations did not show a significant difference in bone formation or in the amount of residual biomaterial. Additionally, the rate of inflammation did not differ significantly between the two groups. The rate of ossification in group A (3 months) was 20.11 ± 11.23%, with 27.67 ± 17.02% in group B (4 months), with no significant difference (*P* = 0.267). The amount of residual biomaterials in group A was 6.82 ± 3.50%, with 7.38 ± 3.04% in group B (*P* = 0.499). The rate of inflammation in group A was 1.7 ± 1.21%, with 1.65 ± 0.67% in group B, with no significant difference (*P* = 0.909). Histological images used in these evaluations showed new bone formation, old bone, and inflammatory responses (Figure 2).

**Table 1:** Comparison of inflammation rate, bone formation, and residual biomaterial percentage between groups A and B

**Group**	**Inflammation**	**Bone formation percentage**	**Residual biomaterial percentage**
Group A	1.7 ± 1.21^a^	20.11 ± 11.23	6.82 ± 3.50
Group B	1.65 ± 0.67	27.67 ± 17.02	7.38 ± 3.04
*P* value	0.909 (NS)	0.267 (NS)	0.499 (NS)

^a^ Data are expressed as mean and SD. NS: Not significant.

## Discussion

 This study evaluated the efficiency of synthetic graft materials in preventing progressive alveolar ridge resorption following tooth extraction. Maintaining the extraction socket with graft material promotes new bone formation without an inflammatory response. The ideal graft material should be osteoconductive, osteoinductive, and highly biocompatible, have an appropriate absorption rate, and be easy to apply.

 In the present study, we eliminated many potential misleading variables by 1) using the exact source of bone grafts for both groups, 2) selecting extraction sockets with a minimum length of 10 mm and a root angle matching the desired final implant position, reducing the risk of native bone removal during biopsy and ensuring accurate histological analysis of bone formation, and 3) employing an acrylic stent to locate the extraction site for consistent biopsy and bone sampling.^13^

 Extensive variations in new bone formation, residual graft material rate, and connective tissue have been reported using various graft materials and ridge preservation techniques. This difference can be influenced by multiple factors such as the status of periodontal disease before extraction, single- and multi-rooted teeth in one study, the size of the extracted tooth socket, the presence or absence of bone fenestration or dehiscence, trauma during tooth extraction, damage to periodontal structures before extracting the tooth, and the angle of the core biopsy relative to the angle of the tooth, etc.^16^

 However, in our study, only single-rooted teeth with the least trauma were extracted, and the extraction sockets were examined for dehiscence or fenestration. No teeth with severe periodontitis were included.

 Leventis et al,^16^ in a study on βTCP graft material, reported a bone regeneration rate of 24.4 ± 7.9% and a residual biomaterial content of 12.9 ± 7.7% after 4 months, which is slightly lower than the 4-month group in our study. Although the sampling time was similar, differences in extraction site location (molar region vs. interdental septa) and carrier material likely contributed to the variation in results.

 The HA/βTCP used in this study contains 70% TCP and 30% hydroxyapatite coating. βTCP material exhibits higher absorption properties due to its high biocompatibility and structural similarity to the constituent materials of bones and teeth. The percentage of residual biomaterials calculated in this study at 3 and 4 months indicates a higher absorption rate. However, to improve osteoconductivity, it is recommended that its absorption rate be slowed down, combined with additional adsorbents, so that osteoblasts can be placed within the scaffold created by the graft for bone formation. This material has proper porosity to allow blood vessels to invade and to release nutrients from the surrounding tissues. Its surface enables the bone to adhere and express the ossification phenotype.

 In a study by Kato et al,^17^ the extraction sockets filled with βTCP collagen showed significantly more newly formed bone than those filled with BIO-OSS collagen at six weeks. Additionally, the rate of bone formation in the tenth week in sockets filled with βTCP collagen was about three times higher than in sockets filled with BIO-OSS collagen. These findings demonstrated that βTCP collagen has better osteoconductivity than BIO-OSS collagen.

 Compared with the previous study by Moghaddas et al,^14^ which used cortical FDBA as the graft material, the current study used HA. Despite this difference in materials, the methodologies of both studies were similar. Our findings are consistent with those of Moghaddas et al,^14^ who examined the impact of FDBA on extraction sockets. Similar to their findings, our study showed that bone parameters improved over time, though differences across time intervals were not statistically significant. This suggests that for single-rooted tooth extractions requiring delayed implant placement, shorter healing periods may still be adequate for implant placement without compromising bone quality. Furthermore, consistent with the results of Moghaddas et al,^14^ our study also found no significant differences in bone formation, residual biomaterial, or inflammatory response between the groups. These findings suggest that despite differences in biomaterials, similar ossification and healing outcomes can be achieved, offering flexibility in clinical decision-making for delayed implant placement.

 Whetman and Mealey^13^ demonstrated that DFDBA, like βTCP, has high biocompatibility and absorption capacity. Their findings on bone formation and residual biomaterials align with our study, although their results indicated a higher rate of bone formation.

 The results reported by Yun et al^10^ indicated that BCP with 30% hydroxyapatite, combined with rhBMP-2, showed better bone formation and space maintenance, especially after 8 weeks, than BioOss. However, more research was recommended due to the limited sample size.^15^

 A study by Dahlin et al^11^ evaluated bone regeneration following the use of the new BCP (BCP 1) that combines 90% β-TCP granular substrates and 10% HA compared to BCP without particulate substrates (BCP 2), which consists of 40% β-TCP and 60% HA, and protein-free mineralized bovine bone (DBBM) through guinea pig mandibular surgery in combination with the GBR method. This study showed that all three materials induced proper bone formation in eight weeks. BCP 1 showed a significantly greater amount of newly formed bone despite having a larger residual bone volume than the other groups. In contrast to the present study, it was reported that βTCP, due to its faster absorption, provided more space within the hydroxyapatite scaffold and accelerated bone formation. In this study, occlusion was performed in the molar area.

 Although collecting bone samples with a trephine bur is a standard method, it may affect the boundaries of tissue samples. For this purpose, the middle of the tissue sample was used in this study. Despite its numerous advantages, histomorphometric evaluation of microscopic sections imposes limitations on interpreting histological sections of reconstructed bone areas due to their two-dimensional representation of three-dimensional space.^18^ Therefore, in addition to the effect of biological factors on the thickness of bony trabeculae, technical issues can explain the differences between study results, such as preparing sections relative to the longitudinal axis of the defect (vertical or parallel), which are quite effective in the obtained microscopic view. Also, the methods for obtaining bone cores in human studies are different.^19^ According to Hong et al,^12^ the percentage of newly formed bone in the control group (no graft) was significantly higher compared to the graft groups in all healing periods. The amount of newly formed bone from HA and BCP increased over time, while the percentage of residual biomaterials showed different patterns; it decreased in BCP, while minimal change was observed in HA. Newly formed bone by β-TCP showed the smallest fraction compared to other graft groups at 2 and 4 weeks; however, it increased significantly during week 8. The residual biomaterial of β-TCP was lower than that of HA and BCP throughout the treatment period. The number of multinucleated cells was higher in BCP and β-TCP, followed by HA, and finally, the lowest was in the control group. In this study, bone samples were taken from apical, middle, and coronal areas. In our study, it was taken only from the middle part.

 The present study observed adult lamellar bone in both the case and control samples. All the samples showed features of newly formed bone, including neovascularization and osteocytes within lacunae. Osteoclasts and reversible lines indicated bone remodeling. The amount of residual biomaterial and connective tissue varies across studies and depends on several factors, including the surgical procedure, the type of graft, and the recovery period.

 In this study, graft materials were surgically inserted without flap retraction in all patients. The extraction socket was sealed first with a collagen sponge (Autoplug), and a suture was placed to hold the graft material. Retracting the periosteum from the dense buccal bone to create a mucoperiosteal flap can reduce blood flow to the exposed bone, activate osteoclasts, and eventually lead to bone destruction. This method is also associated with greater patient satisfaction, reduced costs and time during surgery, and, more importantly, reduced risk of mucogingival junction displacement. It also helps form keratinized soft tissue in the grafted area.^20^ In this study, the grafted sockets healed correctly.

 The rationale for selecting a one-month interval was to determine whether comparable bone formation could be achieved at an earlier stage, potentially reducing overall treatment time and enabling earlier implant placement. During the healing process, bone remodeling is dynamic; previous studies have shown that between the third and fourth months, β-TCP undergoes further resorption, and newly formed bone becomes more mature and mineralized, increasing in density and strength.^13,16,21^ Therefore, evaluating this time frame is clinically relevant for determining the optimal timing of implant placement while balancing efficacy and patient convenience.

 Although the statistical analysis did not show a significant difference in bone formation between the 3- and 4-month groups, clinical observations during core biopsy revealed differences in bone density between some samples. The lower density and softer bone appeared more pronounced in group A, and Whetman and Mealey^13^ also recommended spending more time placing the graft.

 Parameters of connective tissue healing and residual graft material are consistent with other studies on BCP composition. Studies have shown that significant absorption of β-TCP particles is expected within 3–6 months.^17^

 The amount of newly formed bone in different studies is the same as in the present study. In the study by Kakar et al,^21^ over 5.2 ± 2 months, the bone formation rate in 15 extraction sockets treated with BCP was 21.34 ± 9.14%, consistent with group A in the present study. Brkovic et al^22^ showed that, over 9 months of histomorphometric analysis, the amount of newly formed bone and the remaining graft material was 62.6% and 16.3%, respectively. It is possible to reduce the absorption rate of β-TCP substance and use its prolonged effects in the bone formation process by incorporating a percentage of hydroxyapatite into the rapidly absorbing β-TCP. In Kakar and colleagues’ study,^21^ histomorphometric analyses showed that the residual biomaterial remaining during 5.2 ± 2 months was 26.19 ± 9.38%, similar to bone formation levels in the rabbit calvarial defect in Schmidlin’s study. The drawbacks of this study were the non-uniform time interval between taking biopsies from samples, and the number of samples was 15.^8^ Similarly, one of the limitations of the present study was the difficulty in finding patients who met all the inclusion criteria. Additionally, the study’s duration affected patient cooperation, resulting in the loss of some samples. It is recommended to conduct studies with larger sample sizes to enhance the generalizability of the results.

## Conclusion

 HA/βCP bone graft material (OSTEON II) appears to be an effective material for preserving socket dimensions following tooth extraction. It offers a reliable alternative to autogenous bone grafts for maintaining bone structure. Considering the limitations of this study and the lack of statistically significant differences in bone parameters between 3- and 4-month healing periods, early re-entry procedures may be feasible. Further long-term studies with larger sample sizes are recommended to confirm these results.

## Competing Interests

 The authors declare that they have no competing interests regarding authorship and/or publications of this paper.

## Data Availability

 The data that support the findings of this study are available from the corresponding author upon request. All relevant data related to the methodology and results can be provided as supplementary material or through an appropriate data repository upon request.

## Ethical Approval

 This study was approved by Tehran Islamic Azad University, Dental Branch, Research Ethics Committees with Approval ID:IR.IAU.DENTAL.REC.1396,15 and Iranian Registry of Clinical Trials IRCTID: IRCT20180714040460N2.
